# Piceatannol SNEDDS Attenuates Estradiol-Induced Endometrial Hyperplasia in Rats by Modulation of NF-κB and Nrf2/HO-1 Axes

**DOI:** 10.3390/nu14091891

**Published:** 2022-04-30

**Authors:** Lenah S. Binmahfouz, Basma G. Eid, Amina M. Bagher, Rasheed A. Shaik, Najlaa S. Binmahfouz, Ashraf B. Abdel-Naim

**Affiliations:** 1 Department of Pharmacology and Toxicology, Faculty of Pharmacy, King Abdulaziz University, Jeddah 21589, Saudi Arabia; beid@kau.edu.sa (B.G.E.); abagher@kau.edu.sa (A.M.B.); rashaikh1@kau.edu.sa (R.A.S.); aaabdulalrahman1@kau.edu.sa (A.B.A.-N.); 2 Department of Anatomical Histopathology, East Jeddah General Hospital, Jeddah 22253, Saudi Arabia; nahmed-bin-mahfuz@moh.gov.sa

**Keywords:** estradiol benzoate, endometrial hyperplasia, oxidative stress, inflammation, piceatannol

## Abstract

Endometrial hyperplasia (EH) is the most common risk factor for endometrial malignancy in females. The pathogenesis of EH has been directly linked to uterine inflammation, which can result in abnormal cell division and decreased apoptosis. Piceatannol (PIC), a natural polyphenolic stilbene, is known to exert anti-inflammatory, antioxidant and anti-proliferative activities. The aim of the present study was to examine the potential preventive role of PIC in estradiol benzoate (EB)-induced EH in rats. A self-nanoemulsifying drug delivery system (SNEDDS) was prepared to improve the solubility of the PIC. Therefore, thirty female Wistar rats were divided into five groups: (1) control, (2) PIC SNEDDS (10 mg/kg), (3) EB (0.6 mg/kg), (4) EB + PIC SNEDDS (5 mg/kg) and (5) EB + PIC SNEDDS (10 mg/kg). The administration of PIC SNEDDS prevented EB-induced increases in uterine weights and histopathological changes. Additionally, it displayed pro-apoptotic and antioxidant activity in the endometrium. Immunohistochemical staining of uterine sections co-treated with PIC SNEDDS showed significantly decreased expression of tumor necrosis factor-alpha (TNF-α), interleukin-6 (IL-6) and nuclear transcription factor-kappa B (NF-κB). This anti-inflammatory effect was further confirmed by a significant increase in Nrf2 and heme oxygenase-1 (HO-1) expression. These results indicate that SNEDDS nanoformulation of PIC possesses protective effects against experimentally induced EH.

## 1. Introduction

Endometrial hyperplasia (EH) is defined as the irregular proliferation of endometrial cells [1]. Several studies have confirmed that continuous stimulation of unopposed estrogen in the endometrial glandular epithelium plays a significant role in the pathogenesis of EH [2,3]. Common causes for excessive estrogen exposure may include obesity, anovulatory cycles (polycystic ovarian syndrome and menopause), hormone replacement therapy and tamoxifen (for breast cancer) [4]. Patients with EH mainly present with abnormal uterine bleeding. In persistent long-term cases, EH may progress to endometrial cancer (EC), the most common gynaecologic cancer in females [5]. It has been estimated that the incidence of EH is three times the number of EC cases [6].

According to the World Health Organization classification, there are two types of EH: (i) hyperplasia without atypia, characterized by minimal glandular crowding in the endometrium that is largely self-limited, and (ii) atypical hyperplasia, characterized by complex endometrial glandular crowding, cytological atypia and a high risk of progression to EC [7,8]. Typically, hyperplasia without atypia is managed with progestogen therapy, whereas the gold standard treatment for women with atypical hyperplasia after childbearing age is a total hysterectomy [9,10]. However, this approach is often unacceptable for younger women. Therefore, non-surgical alternative treatments are still in demand [11,12].

The underlying pathological effects of endometrial estrogen exposure are mediated by inflammation and oxidative stress. Uterine inflammation results in abnormal cell division and decreased apoptosis [13]. Histological evidence of inflammation has been reported in different models of EH [14,15]. Patients with EH secrete elevated levels of pro-inflammatory cytokines, such as tumor necrosis factor-α (TNF-α) and interleukins (IL-1β and IL-6) [14]. In addition, immunohistochemical studies on human endometrial specimens showed that members of the nuclear transcription factor-kappa B (NF-κB) family were expressed in EH and carcinoma [16]. Moreover, it has been reported that estrogen and its metabolites act as pro-oxidants and induce the generation of reactive oxygen species (ROS) [17,18]. In turn, ROS stimulates the phosphorylation of different kinases that activate nuclear transcription factors (such as NF-κB and NRF1), which participate in the progression of cancer [18].

Piceatannol (PIC) and resveratrol are natural stilbenes (polyphenols) found in different foods, such as passion fruit seeds, grapes, peanuts and white tea [19]. In humans, cytochrome p450 (cyp1B1) metabolizes resveratrol into PIC, which is a more metabolically stable analog [20]. Because of its beneficial effects on numerous diseases, PIC has raised interest within the medical community [21]. It has been proven that PIC exhibits a broad spectrum of pharmacological activities against different types of medical conditions, including cardiovascular diseases, diabetes, benign prostatic hyperplasia (BPH) and cancer [22,23,24,25]. Piceatannol is a typical pleiotropic agent as it regulates different cellular processes, such as apoptosis, proliferation and oxygen radical formation [26]. In the literature, PIC has shown much higher free radical scavenging activity than resveratrol [27]. Furthermore, PIC reported anti-inflammatory effects by suppressing the levels of pro-inflammatory cytokines TNF-α and IL-6 in macrophages with higher potency than resveratrol [28]. PIC also inhibited TNF-α-induced NF-κB activation in several cell types, which plays a pivotal role as a transcriptional regulator in response to cellular stress [22]. This suggests that PIC could potentially possess anti-inflammatory and antioxidant activities in EH.

However, PIC suffers low solubility and bioavailability compared to resveratrol [20]. Hence, nanodrug formulations have been employed in different studies to improve the safety, efficacy and pharmacokinetic or pharmacodynamic properties of PIC [25,26,29]. In the current study, the aim was to investigate the preventive effects of PIC nanoparticles against EB-induced EH in female rats and to explore the potential underlying mechanism.

## 2. Materials and Methods

### 2.1. Chemicals

Estradiol benzoate (beta-estradiol 3-benzoate) was purchased from Sigma-Aldrich (Schnelldorf, Germany) via their supplier Bayouni Trading Co. (Riyadh, Saudi Arabia). Piceatannol (98% purity) was obtained from Beijing Yibai Biotechnology Co., Ltd. (Beijing, China). All remaining chemicals were of the highest commercial analytical grade.

### 2.2. Animals

A total of 30 eight-week-old female Wistar rats (150–190 g) were purchased from the Faculty of Pharmacy, King Abdulaziz University, Jeddah. The rodents were housed in a 12 h light-dark cycle at 22 ± 2 °C temperature and fed ad libitum with freely accessible water for one week of acclimatization in our animal facility before the experiment. All procedures were approved by the Research Ethics Committee at the Faculty of Pharmacy, King Abdulaziz University, Jeddah, Saudi Arabia (Reference No. PH-1442-55). The guidelines from the National Institute of Health for the care and use of laboratory animals (NIH Publications No. 8023, revised 1978) were followed in all animal experiments.

### 2.3. Preparation of PIC Self-Nanoemulsifying Drug Delivery System (SNEDDS)

A SNEDDS formulation was prepared by combining polyethylene glycol 200 (surfactant) and Tween 80 (cosurfactant) with oleic acid at a ratio of 4:4:2. After that, 50 mg PIC was mixed with the components using a vortex mixer for 5 min at room temperature totaling 1 g of PIC-SNEDDS. The PIC-SNEDDS formulation was further sonicated in water for 50 s using a sonics probe sonicator (20 kHz, 1500 W, Sonics, Newtown, CT, USA) to allow for complete dissolution. The particle size of the resultant nanoformulation was determined using the dynamic light scattering (DLS) technique integrated into the Zetasizer analyzer (Malvern Instrument, Worcestershire, UK). For that, 100 μL of the PIC-SNEDDS formulation was diluted with 10 mL of 0.1 N HCL in a screw-capped glass vial and measured at 25 °C. The average particle size was 75 ± 2.1 nm.

### 2.4. Acute Toxicity Study

A single oral dose of PIC SNEDDS (2000 mg/kg) was administered orally to three experimental female rats and caused no mortality. According to the guidelines described by the Organization for Economic Co-operation and Development (OECD), the same procedure was repeated after 24 h using three additional rats and led to the same result (guideline no. 423, 2002).

### 2.5. Study Design and Animal Treatment

Thirty female rats were randomly divided into five different groups (6/group). Group 1 (control group) received plain vehicle of the SNEDDS preparation (10 mL/kg, PO) once daily by oral gavage and injected with corn oil (1 mL/kg, SC) three times per week. Group 2 received PIC SNEDDS at a dose of 10 mg/kg (PO) once daily by oral gavage and a corn oil injection (1 mL/kg, SC). Group 3 received EB in corn oil (0.6 mg/kg, SC) three times per week to induce EH and plain vehicle of the SNEDDS preparation (10 mL/kg, PO) once daily. Groups 4 and 5 received PIC SNEDDS at a dose of 5 or 10 mg/kg (PO, respectively) once daily concomitantly with EB (0.6 mg/kg, SC) three times per week. The selected doses of PIC are based on an initial pilot study and are consistent with previously published literature [24,25]. The duration of the experiment was four consecutive weeks.

At the end of the study, rats were weighed then anesthetized using ketamine (80 mg/kg, IP) and xylazine (80 mg/kg, IP) followed by cervical dislocation and the uteri were harvested. After the removal of fat and connective tissues, wet uteri from all the groups were visually examined, then slit to remove extra water and weighed immediately. The two horns were treated separately. The middle part of one horn was fixed in 10% neutral buffered formalin for histological and immunohistochemical examination. The other horn was snap-frozen in liquid nitrogen and thereafter stored at −80 °C until used for real-time polymerase chain reaction (RT-PCR) and other biochemical analyses.

### 2.6. Histological Examination

The formalin-fixed uterine tissues were subjected to the paraffin embedding process. Subsequently, the paraffin-embedded sections (5 μm) were stained using hematoxylin and eosin (H&E) for routine histological examination. The slides were examined using an electric light microscope (Carl Zeiss Axiostar plus, Oberkochen, Germany), and the endometrial epithelium thickness was measured using Image J software (1.46a, NIH, Bethesda, MD, USA).

### 2.7. Assessment of Apoptotic Markers

#### 2.7.1. mRNA Expression of Bax and Bcl-2

The mRNA expression of Bax and Bcl-2 was evaluated using an RT-PCR assay. First, uterine tissues from all different groups were homogenized using an ultrasonic probe. Total RNA was extracted from the homogenized samples using the RNeasy^®^ Mini Kit (Qiagen, Valencia, CA, USA), and then its purity and concentration were measured using a spectrophotometer. cDNAs were generated from total RNA using the Reverse Transcription Kit (Applied Biosystems, CA, USA). After that, RT-PCR reactions were carried out using a Taq PCR Master Mix Kit (Qiagen, CA, USA). The β-actin was used as the housekeeping gene. The forward and reverse primer sets for each gene were ordered from Sigma-Aldrich (Gillingham, UK) and their sequence is shown in Table 1.

#### 2.7.2. Caspase-3 Concentration by ElISA

The concentration of the cleaved caspase-3 in the uterine tissue homogenates was determined using PathScan^®^ Cleaved Caspase-3 (Asp175) Sandwitch ELISA Kit (Catalog # 7190, Cell Signaling Technology, Inc., Danvers, MA, USA).

### 2.8. Oxidative Stress Biomarkers Assessment

The concentrations of malondialdehyde (MDA) lipid peroxidation, Superoxide Dismutase (SOD) and Catalase (CAT) in the uterine homogenates were measured using commercially available kits with product numbers: MD-2528, SD-2521 and CA-2516 (Biodiagnostics, Cairo, Egypt) following the manufacturer’s protocols.

### 2.9. Immunohistochemical Staining

Immunohistochemical detection of NF-κB (p65), TNF-α, IL-6, Nrf2 and heme oxygenase-1 (HO-1) was performed on uterine sections embedded in paraffin. Deparaffinized sections were gradually rehydrated using ethanol, then boiled in 10 mM sodium citrate buffer (pH 6.0) for 5 min, rinsed with phosphate-buffered saline and processed for immunostaining. The slides were blocked in 5% bovine serum albumin (BSA) in Tris-base buffered saline (TBS) for 1 h at room temperature. Sections were then incubated overnight at 4 °C with one of the following primary antibodies: IL-6 (Catalog # ab271269), TNF-α (Catalog # ab205587), NF-κB (p65) (Catalog # ab16502), Nrf2 (Catalog # ab207233) or HO-1 (Catalog # ab189491) (ABCAM, Cambridge, UK). The next day, the sections were washed with TBS and then incubated with the corresponding secondary antibody. The slides were visualized using a light microscope and photomicrographs were captured with a CCD camera.

The immunohistochemically stained sections were evaluated semi-quantitatively using the H-score [30]. Briefly, five different fields were randomly chosen from at least three selected slides. For each field, a percentage was given for the positive-stained cells. Then, the intensity of the brown staining was categorized as 0 (negative), 1 (weak), 2 (intermediate) and 3 (strong). The H-score value was calculated using the following formula: (% of cells stained at intensity category 1 × 1) + (% of cells stained at intensity category 2 × 2) + (% of cells stained at intensity category 3 × 3). The results were presented graphically.

### 2.10. Data Analysis

Data are expressed as mean ± standard deviation (S.D) and screened for normal distribution using the Shapiro–Wilk’s test. Statistical significance was determined using one-way analysis of variance (ANOVA) followed by Tukey’s post hoc test utilizing GraphPad Prism software version 8 (La Jolla, CA, USA) as appropriate. The Kruskal–Wallis test and Mann–Whitney U-test were used for non-parametric data. *p* values < 0.05 were considered significant.

## 3. Results

### 3.1. Gross Examination

**Figure 1:**
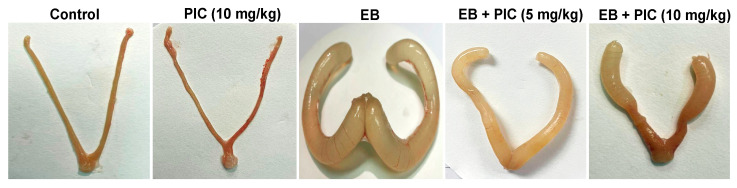
Effect of piceatannol SNEDDS on uterine morphology in rats treated with estradiol benzoate. Gross images of rat uteri collected after four weeks of treatment with estradiol benzoate (0.6 mg/kg) in the absence or presence of piceatannol SNEDDS (5 or 10 mg/kg). EB is estradiol benzoate, PIC is piceatannol, SNEDDS is self-nanoemulsifying drug delivery system.

The morphology of the uterine tissues was assessed after exposure to 0.6 mg/kg EB with or without PIC SNEDDS (5 or 10 mg/kg) for four consecutive weeks (Figure 1). Upon visual inspection, the uteri of the control and PIC (10 mg/kg) groups showed no dilation of the horn or any other abnormalities. Uteri of rats treated with EB (0.6 mg/kg) contained turbid and thick fluid that resulted in marked dilation of the uterine horn. Concomitant administration of PIC SNEDDS (5 and 10 mg/kg) showed uteri containing only serous fluid that moderately dilated the uterine horn less than that observed in the EB group.

### 3.2. Body and Uterine Weights

Piceatannol SNEDDS (10 mg/kg) treatment alone did not adversely affect relative uterine weight when compared to the control, as shown in Table 2. However, EB injection significantly increased relative uterine weight by 224% compared to the control group. Both doses of PIC SNEDDS (5 and 10 mg/kg) significantly decreased the relative uterine weight gain by about 46% (*p* > 0.05). However, this effect on uterine weight was not dose-dependent.

### 3.3. Histopathological Examination

**Figure 2:**
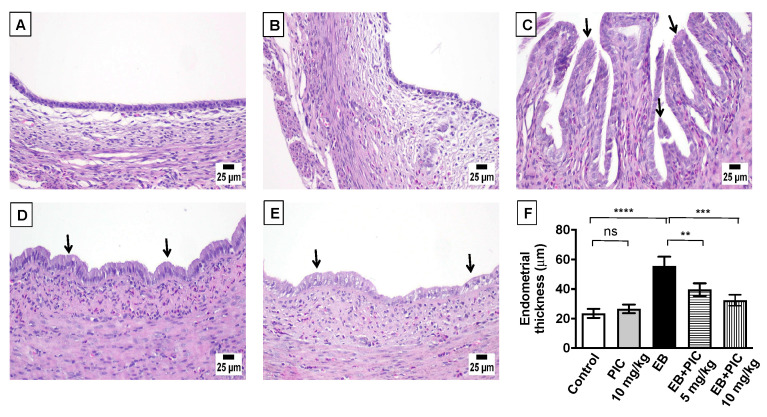
Effect of piceatannol SNEDDS on uterine histopathology in EB-induced EH in rats. Shown are uterus sections stained with Hematoxylin and eosin (H&E) from the following groups: (**A**) control group showing the normal histoarchitecture of the uterus; (**B**) piceatannol SNEDDS (PIC) 10 mg/kg group with no observable histological changes; (**C**) estradiol benzoate (EB) 0.6 mg/kg group showing increased endometrium thickness with hyperplasia and intraluminal papillary projections; (**D**) EB group co-treated with PIC (5 mg/kg) showing reduction in hyperplasia and projections; (**E**) EB group co-treated with PIC (10 mg/kg) showing a clear reduction in hyperplasia and projections. Arrows point to intraluminal papillary projections. Panel (**F**) represents a graphical presenation of endometrial thickness. Dara are expressed as mean ± S.D (*n* = 6). ns = not significant, ** *p* < 0.01, *** *p* < 0.001 and **** *p* < 0.0001 by one-way ANOVA with Tukey’s post hoc test.

Histopathological examination (H&E staining) of uterine tissues collected from control and PIC SNEDDS alone (10 mg/kg)-treated animals revealed the normal architecture of the endometrium with a normal epithelial layer (Figure 2A,B). On the contrary, EB injection resulted in marked EH with epithelial proliferation and intraluminal papillary projections as shown in Figure 2C. Co-treatment with PIC SNEDDS (5 and 10 mg/kg) resulted in much less hyperplastic growth and projections induced by EB in a dose-related manner (Figure 2D,E). These data were further confirmed by evaluating the endometrial thickness. As depicted in Figure 2F, the endometrium in the EB group was thickened by 136% in comparison to the control group. At doses of 5 and 10 mg/kg PIC SNEDDS, endometrial thickness was significantly decreased by 29% and 42%, respectively, compared to the EB group.

### 3.4. Assessment of mRNA Expression of Bax and Bcl-2

**Figure 3:**
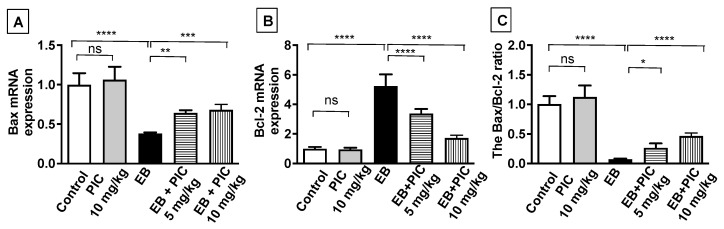
The effect of PIC SNEDDS treatment on the mRNA expression of Bax (**A**) and Bcl-2 (**B**) and the Bax/Bcl-2 ratio (**C**) in the uterine tissue. Data are shown as Mean ± S.D (*n* = 6). ns = not significant, * *p* < 0.05, ** *p* < 0.01, *** *p* < 0.001 and **** *p* < 0.0001 by one-way ANOVA with Tukey’s post hoc test.

To assess the effect of PIC SNEDDS on apoptosis, Bax, Bcl-2 and caspase-3 were measured by different techniques. First, the mRNA expression level of the apoptotic markers Bax and Bcl-2 was assessed by the RT-PCR technique (Figure 3). No significant changes in both Bax and Bcl-2 expression levels were observed in animals treated with PIC SNEDDS alone (10 mg/kg) relative to the control. In contrast, a significant decrease in Bax mRNA expression by 63% was observed in the EB-treated group, while the mRNA expression of Bcl-2 increased significantly by 450% in comparison to the control. Interestingly, PIC SNEDDS (5 and 10 mg/kg) co-administration resulted in a significant elevation in Bax mRNA levels by 64% and 82%, respectively, and a marked decrease in Bcl-2 mRNA levels by 36% and 73%, respectively, relative to the EB-treated group. Furthermore, the Bax/Bcl-2 ratio was markedly decreased by 93% in the EB-treated group relative to the control group. Importantly, the lower dose of PIC SNEDDS (5 mg/kg) significantly elevated the ratio of Bax/Bcl-2 by 265% relative to EB, while the value was significantly increased by 540% after treatment with the higher dose of PIC SNEDDS (10 mg/kg).

### 3.5. Caspase-3

**Figure 4:**
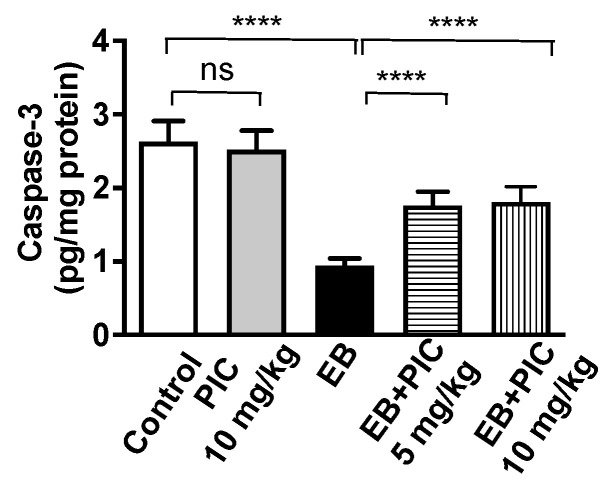
Effect of PIC SNEDDS treatment on caspase-3 concentration in the uterine tissue. Data are shown as Mean ± S.D (*n* = 6). ns = not significant and **** *p* < 0.0001 by one-way ANOVA with Tukey’s post hoc test.

As shown in Figure 4, no significant change in caspace-3 content was observed in PIC (10 mg/kg) only group as compared to the control group. Treatment with EB markedly decreased the content of capsase-3 in uterine tissues by 65% compared to the control group. As expected, PIC SNEDDS co-administration of 5 and 10 mg/kg significantly prevented the decline in caspase-3 content by 87% and 92% as compared to the EB-treated group.

### 3.6. Assessment of Oxidative Stress Markers

The concentration of MDA (lipid peroxidation) and the activity of SOD and CAT in uterine tissues were measured for all groups to evaluate the effects of PIC SNEDDS on oxidative stress. The PIC SNEDDS alone (10 mg/kg) did not affect any of the oxidative stress markers measured (Table 3). However, EB treatment resulted in a significant accumulation of MDA in uterine tissue by 237% relative to the control group. Concomitant treatment of EB with PIC SNEDDS (5 and 10 mg/kg) significantly reduced the MDA concentration by 43% and 53%, respectively, relative to EB-treated rats. On the contrary, the activity of SOD and CAT antioxidant enzymes in the uterine tissue was depleted in the EB-treated group by 46% and 26%, respectively, relative to the control group. Meanwhile, co-treatment with PIC SNEDDS significantly ameliorated SOD depletion and boosted its value by 49% with 5 mg/kg and by 63% with 10 mg/kg when compared to the EB-treated rats. Similarly, CAT exhaustion was reversed by 22% with the lower dose of PIC SNEDDS and by 24% with the higher dose. Hence, PIC SNEDDS antioxidant activity might be responsible for its protective anti-apoptotic role in EH.

### 3.7. Immunohistochemical Determination of Uterine Expression of IL-6, TNF-α, NFκB (p65), HO-1 and Nrf2

**Figure 5:**
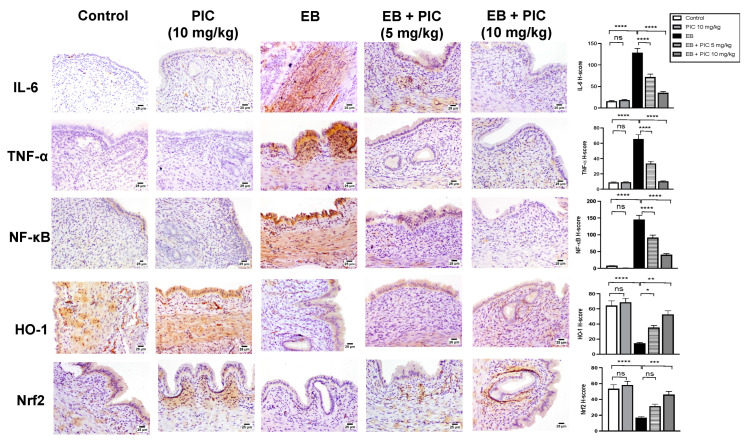
Effect of PIC SNEDDS on EB-induced alterations of IL-6, TNF-α, NFκB, HO-1 and Nrf2 expression in the uterine tissue of rats by immunohistochemical staining. Data presented in bar graphs are the mean of H-scores ± S.D (*n* = 6). ns = not significant, * *p* < 0.05, ** *p* < 0.01, *** *p* < 0.001 and **** *p* < 0.0001.

The anti-inflammatory activity of PIC SNEDDS in EH was investigated. As shown in Figure 5, the control group showed minimal immunostaining for IL-6, TNF-α and NF-κB (p65). Treatment of rats with PIC SNEDDS alone (10 mg/kg) did not show any change in the expression of these inflammatory markers. In contrast, injection of EB significantly enhanced the expression of the inflammatory markers IL-6, TNF-α and NF-κB (in the assessed nuclei) as depicted by the intense brown staining. Quantification of optical densities confirmed the significantly enhanced expression of IL-6, TNF-α and NF-κB (p65) by 186%, 190% and 136%, respectively, compared to the control value. Administration of PIC SNEDDS (5 and 10 mg/kg) resulted in significant protection against the EB-induced increase in the inflammatory markers IL-6, TNF-α and NF-κB (p65). On the other hand, EB treatment significantly decreased the expression levels of HO-1 and Nrf2 in uterine tissue by 42% and 36% compared to the control group. At a dose of 5 mg/kg, PIC SNEDDS had marked protection with regards to the decline in HO-1 and Nrf2 expression and increased their levels by 35% and 27% of the EB values. At the higher dose of 10 mg/kg, PIC SNEDDS resulted in a significant increase in the expression of HO-1 and Nrf2 to almost normal levels.

## 4. Discussions

Endometrial hyperplasia is characterized by excessive proliferation of the endometrial lining, with hyperplastic changes in glandular and stromal structures. Although benign, this condition may progress to EC if left untreated [5]. A reduction in apoptosis, increased proliferation, as well as excessive oxidative stress and inflammation, have all been recognized as significant culprits in the development of EH [14,31]. The currently available treatment modalities for EH are quite invasive and unsatisfactory. Thus, a need to develop new treatment methods is required to limit the risk of EH. A natural polyphenolic stilbene, PIC is a proven antioxidant with anti-inflammatory and anti-proliferative activities [19,22,25,32,33]. Therefore, the current study aimed to investigate the protective role of PIC in an EH rat model.

A metabolite of resveratrol, PIC was reported to have pro-apoptotic and anti-proliferative effects in the human endometrium at high concentrations. Resveratrol has been shown to have potent anti-inflammatory properties in multiple organ injuries by regulating different intracellular signaling pathways, such as PI3K/Akt and HO-1/MAPK [34]. In fact, PIC is more potent than resveratrol [28]. In this study, PIC was formulated as SNEDDS to enhance the oral bioavailability and internal absorption of this poorly soluble lipophilic compound [35]. This preparation is highly nontoxic, as confirmed by the Acute Toxic Class Method reported in OECD guidelines No.423, which revealed that this formulation is Category 5 with LD50 over 2000 mg/kg (Globally Harmonized System of Classification and Labeling of Chemicals).

In the current study, EB was used to induce the EH model. Estradiol benzoate (an estradiol ester) is a prodrug of 17β-estradiol, which is considered a bioidentical and natural form of estrogen [36]. Following administration, EB is readily cleaved in the liver, tissues and blood into estradiol and the natural fatty acid benzoate through esterase enzymes [37]. This lipophilic fatty acid ester moiety provides EB an extended duration when administered intramuscularly or subcutaneously. Hence, EB was used in the current study as a source of estrogen instead of 17β-estradiol. Exposure to 0.6 mg/kg EB for four weeks significantly increased relative uterine weight in female rats compared with the control. The co-treatment of rats with PIC SNEDDS markedly prevented EB-mediated increase in relative uterine weights. Histologically, this was confirmed, since the PIC resulted in an amelioration of the histopathological changes due to EB and resulted in significant improvement in hyperplasia and projections and reduced the endometrial thickness. It should be noted that the higher dose of PIC SNEDDS (10 mg/kg) showed no significant benefit to uterine weights over the lower dose (5 mg/kg). Together, our results suggest that PIC SNEDDS treatment has a potential protective effect on EH.

Normal endometrium is in a delicate balance between apoptosis and cell proliferation. Endometrial apoptosis is regulated by two major genes: Bax and B-cell lymphoma/leukemia-2 (Bcl-2). Bax is regarded as a pro-apoptotic gene (promotes apoptosis), whereas Bcl-2 is considered an anti-apoptotic gene (inhibits apoptosis). Cytochrome c release from the mitochondria is halted by Bcl-2 and stimulated by Bax [38]. Stimulation of cytochrome c causes caspase-3 activation and increased apoptosis. A study conducted by Mirakhor Samani and co-workers showed that lower levels of Bax in frozen endometrial specimens were markedly associated with malignancy [39]. Results from the present study revealed that Bax levels were decreased and Bcl-2 levels were increased after EB injection, leading to a reduced Bax/Bcl-2 ratio in these rats. Indeed, the anti-apoptotic effect of estrogen has been previously reported in the human endometrium by causing down-regulation of Bax and up-regulation of Bcl-2, which lowers cytochrome c levels and activates caspace-3 [40,41]. In contrast, PIC SNEDDS significantly enhanced apoptosis in the endometrium by Bcl-2 suppression and Bax up-regulation, ultimately raising the Bax/Bcl-2 ratio. Furthermore, caspase-3 levels were reduced after EB treatment, whereas PIC co-treatment (5 or 10 mg/kg) ameliorated the decreased caspase-3 levels. Together, a high Bax/Bcl-2 ratio and increased levels of caspase-3 indicate that PIC exhibits pro-apoptotic and anti-proliferative activity in the endometrium.

Due to the structural similarity to the estrogenic agent diethylstilbestrol, PIC has been reported to be a phytoestrogen compound [42]. The binding of phytoestrogens to estrogen receptors depends on alterations in endogenous estrogen concentration. At high estrogen levels, PIC may have antiestrogenic activity by competitively preventing the binding of estrogen to the estrogen receptor alpha [43]. In general, the antiestrogenic activity of the phytoestrogens is evident in presence of estradiol due to their antagonistic actions on ERα. However, the agonistic potency of phytoestrogens is significant at ERβ [44]. This helps to explain the observed anti-proliferative actions of piceatannol against ERβ-induced uterine hyperplasia.

Oxidative stress plays a key role in the pathogenesis of EH [45]. In the present study, PIC co-administration was found to inhibit the lipid peroxidation of the endometrium induced by EB exposure. These lipid peroxides result from the reactivity of lipids with superoxide anion (O^2−^) [46]. β-oxidation ultimately converts these products to MDA [47]. The MDA level was significantly elevated in the endometrium of the rats with EH compared with the control or PIC-treated rats. Co-treatment with PIC ameliorated the increase in MDA. On the other hand, SOD is an enzyme responsible for catalyzing the O^2−^ into hydrogen peroxide (H_2_O_2_). In turn, CAT converts H_2_O_2_ into water [46,48]. We observed that PIC offered protection from raised oxidative stress by reversing SOD and CAT depletion due to EB administration. These results are aligned with the previously reported antioxidant effects of PIC. In vivo, PIC treatment (10 mg/kg) significantly inhibited streptozotocin-induced oxidative stress in a diabetic cardiomyopathy (DCM) rat model, as measured by the decreased MDA concentration in cardiac tissue and serum [24]. Moreover, PIC (10 mg/kg) showed antioxidant effects against cisplatin-induced nephrotoxicity in rats, such as the inhibition of lipid peroxidation and increased SOD and GSH levels [33]. Therefore, PIC may be beneficial for the prevention and treatment of EH.

Immunohistochemical investigation of the uterine tissues revealed an anti-inflammatory effect of PIC on EB-induced EH in rats. The pathology of EH has been linked to increased transcriptional factors (e.g., nuclear NF-κβ) that subsequently activate multiple pro-inflammatory cytokines, such as IL-6 and TNF-α [16]. Pro-inflammatory cytokines may stimulate the expression of genes involved in anti-apoptosis and metastasis [14]. In the current investigation, PIC SNEDDS inhibited the raised expression of IL-6, TNF-α and NF-κβ (p65), suggesting that the anti-inflammatory effects of PIC in EH are mediated through the NF-κβ pathway. This result is consistent with the established anti-inflammatory role of PIC and other stilbenes [49].

To identify the mechanism underlying the anti-inflammatory effect of PIC SNEDDS on the EB-induced NF-κB activation, HO-1 and Nrf2 expression levels were measured by immunostaining. The Nrf2/HO-1 signaling pathway has been receiving wide attention due to its important role in inflammation as a result of NF-κβ activation [50]. Nrf2 binding stabilizes the Kelch-like ECH-associated protein 1. After Nrf2 dissociates from Keap1, it makes its way into the nucleus and binds to antioxidant response elements. In turn, the downstream activation of HO-1 follows, causing relief of inflammation by alternating p65 translocation [50]. Studies on different cell lines, such as bovine endothelial cells and MCF10A epithelial cells, have revealed the ability of PIC to increase HO-1 expression and Nrf2 activation [51,52]. Moreover, this effect was also observed in a diabetic cardiomyopathy rat model [24]. A recent study of PIC in a model of BPH also reported similar findings, whereby PIC stimulated Nrf2 protein expression, which decreased due to testosterone administration [25]. In the current study, PIC co-treatment reversed the reduced Nrf2 and HO-1 expression mediated by EB, suggesting that PIC suppresses NF-κB activation via the Nrf2/HO-1 axis. Ultimately, the anti-inflammatory effects exerted by PIC could have contributed significantly to the anti-hyperplastic effect.

Several factors can be considered as limitations to the current study. For example, previous studies have been performed via bilateral ovariectomy two weeks before the induction of EH to exclude the potential influence of ovarian steroid hormones [53]. However, EH was induced in several other studies using the same current experimental model [15,54,55]. The repeated administration of EB (0.6 mg/kg) for four weeks covered at least seven consecutive estrous cycles in the experimental rats and minimized the variations in the endogenous steroid hormone levels [56]. Thus, ovariectomy and cycle synchronization would have almost no or minor impact. Another limitation is the small number of rats in each group (*n* = 6), which might affect the statistical significance of the results. In addition, the acute toxicity study of PIC SNEDDS was first conducted to assess its safety in the current model. The potential sub-acute toxicity of PIC after repeated oral administration in rats is to be explored to assess any possible adverse effects.

## 5. Conclusions

In conclusion, our results suggest that PIC exerts preventive effects against estradiol-induced EH. This is attributed, at least partly, to PIC antioxidant, anti-inflammatory and proapoptotic activities, as well as the modulation of NF-κB and Nrf2/HO-1 signaling.

## Figures and Tables

**Table 1:** Primers sequences used for the analysis of gene expression.

Target Gene	Primer Sequence	Gene Bank Accession Number
Bax	Forward: 5′-CCTGAGCTGACCTTGGAGCA-3′ Reverse: 5′-GGTGGTTGCCCTTTTCTACT-3′	U32098.1
Bcl-2	Forward: 5′-TGATAACCGGGAGATCGTGA-3′ Reverse: 5′-AAAGCACATCCAATAAAAAGC-3′	NM_016993.1
β-actin	Forward: 5′-CTAAGGCCAACCGTGAAAAG-3′ Reverse: 5′-GCCTGGATGGCTACGTACA-3′	NM_031144.3

Data were expressed in the Cycle threshold (Ct). The relative quantification (RQ) of each gene to β-actin was quantified based on the delta-delta Ct (ΔΔCt) method calculation.

**Table 2:** Effect of PIC SNEDDS (5 and 10 mg/kg) on uterine weight in EB-induced EH in rats.

Group	Uterine Weight (g)	Body Weight (g)	Relative Uterine Weight (× 10^3^)
Control	0.39 ± 0.06	188.6 ± 9.09	2.29 ± 0.10
PIC (10 mg/kg)	0.43 ± 0.12	191.0 ± 8.86	2.62 ± 0.12
EB	0.97 ± 0.12	190.4 ± 14.38	7.45 ± 0.54 ^a,b^
EB + PIC (5 mg/kg)	0.80 ± 0.05	181.4 ± 12.97	4.04 ± 0.29 ^a,b,c^
EB + PIC (10 mg/kg)	0.70 ± 0.11	170.0 ± 13.50	4.07 ± 0.32 ^a,b,c^

Results are shown as mean ± S.D (*n* = 6). To calculate the relative uterine weight, the uterine weight (g) was divided by the bodyweight (g) of the rat and multiplied by 103. Results are considered significantly different when *p* < 0.05 by one-way ANOVA with Tukey’s post hoc test. ^a^ Significantly different from control group, ^b^ Significantly different from PIC (10 mg/kg) group, and ^c^ Significantly different from EB group. PIC is piceatannol SNEDDS-treated group, EB is estradiol benzoate-treated group, EB + PIC is estradiol benzoate and piceatannol SNEDDS treated group.

**Table 3:** Effect of PIC SNEDDS (5 and 10 mg/kg) on uterus oxidative status in EB induced EH in rats.

Group	MDA (nmol/mg Protein)	SOD (U/mg Protein)	CAT (U/mg Protein)
Control	0.51 ± 0.075	36.44 ± 4.51	2.44 ± 0.034
PIC (10 mg/kg)	0.46 ± 0.063	39.92 ± 4.70	2.33 ± 0.287
EB	1.72 ± 0.21 ^a,b^	19.82 ± 2.20 ^a,b^	1.81 ± 0.210 ^a,b^
EB + PIC (5 mg/kg)	1.01 ± 0.12 ^a,b,c^	29.57 ± 3.54 ^a,b,c^	2.21 ± 0.240 ^c^
EB + PIC (10 mg/kg)	0.82 ± 0.094 ^a,b,c^	32.35 ± 3.30 ^a,b,c^	2.25 ± 0.290 ^c^

MDA is malondialdehyde, SOD is superoxide dismutase, CAT is catalase. Results are shown as Mean ± S.D (*n* = 6). Results are considered significantly different when *p* < 0.05. ^a^ Significantly different from control group, ^b^ Significantly different from PIC (10 mg/kg) group, and ^c^ Significantly different from EB group. PIC is piceatannol SNEDDS-treated group, EB is estradiol benzoate-treated group, EB + PIC is estradiol benzoate and piceatannol SNEDDS treated group.

## Data Availability

The authors declare that the data supporting the findings of this study are presented in the manuscript. The primer sequences obtained in this study for Bax, Bcl-2 and beta-actin are listed in Table 1 under the accession number U32098.1, NM_016993.1, and NM_031144.3, respectively.
